# Adsorption of vancomycin, gentamycin, ciprofloxacin and tygecycline on the filters in continuous renal replacement therapy circuits: in full blood in vitro study

**DOI:** 10.1007/s10047-020-01214-8

**Published:** 2020-10-08

**Authors:** Dariusz Onichimowski, Krzysztof Nosek, Hubert Ziółkowski, Jerzy Jaroszewski, Aleksandra Pawlos, Mirosław Czuczwar

**Affiliations:** 1 Department of Anaesthesiology and Intensive Therapy, Faculty of Medicine, University of Warmia and Mazury, Al. Warszawska 30, 11-082 Olsztyn, Poland; 2 Department of Pharmacology and Toxicology, Faculty of Veterinary Medicine, University of Warmia and Mazury, Oczapowskiego 13, 10-719 Olsztyn, Poland; 3 Department of Pharmacology and Toxicology, Faculty of Medicine, University of Warmia and Mazury, Al. Warszawska 30, 10-082 Olsztyn, Poland; 4 2nd Department of Anaesthesiology and Intensive Therapy, Medical Univeristy of Lublin, Staszica 16, 20-081 Lublin, Poland

**Keywords:** Vancomycin, Gentamycin, Ciprofloxacin, Tigecycline, Adsorption

## Abstract

The aim of this study was to assess the in vitro adsorption of antibiotics: vancomycin, gentamicin, ciprofloxacin and tigecycline on both polyethyleneimine-treated polyacrylonitrile membrane of AN69ST filter and polysulfone membrane of AV1000 filter using porcine blood as a model close to in vivo conditions. The porcine blood with antibiotic dissolved in it was pumped into hemofiltration circuit (with AN69ST or AV1000 filter), ultrafiltration fluid was continuously returned to the reservoir containing blood with antibiotic. Blood samples to determine antibiotic concentrations were taken at minutes 0, 5, 15, 30, 45, 60, 90 and 120 from the pre- blood pump of the hemofiltration circuit. To assess possible spontaneous degradation of the drug in the solution there was an additional reservoir prepared for each antibiotic, containing blood with the drug, which was not connected to the circuit. In the case of vancomycin, ciprofloxacine and tigecycline, a statistically significant decrease in the drug concentration in the hemofiltration circuit in comparison to initial value as well as to the concentrations in the control blood was observed, both for polyacrylonitrile and plolysulfone membrane. In the case of gentamicin, significant adsorption was noted only on polyacrylonitrile membrane. Our studies demonstrated that in full blood adsorption of antibiotics may be big enough to be of clinical significance. In particular in the case of polyacrylonitrile membrane.

## Introduction

Optimum antibiotic dosing in the treatment of severe infections and septic shock is of key importance for achieving a therapeutic success; it remains, however, a major challenge in intensive therapy units. This is due to the complexity and variability of antibiotic pharmacokinetics in critically ill patients as well as increasing antimicrobial resistance, but also due to the use of medical technologies which have an effect on elimination of antibiotics from blood. What has been seen over the recent years is a growing number of intensive therapy unit patients with septic shock-induced multiorgan failure who also develop acute kidney injury (AKI). The incidence of AKI in patients with septic shock reaches 65% [1]. Consequently, there is a large group of patients who require continuous renal replacement therapy (CRRT). It must be noted, however, that during this procedure many drugs may be eliminated, including antibiotics which are the cornerstone in the treatment of sepsis.

The mechanisms of antibiotic elimination during CRRT include diffusion, ultrafiltration and adsorption on filter membranes [2]. Of these, ultrafiltration and diffusion have been the subject of many studies, with considerably fewer papers on adsorption published. As of date, the highest adsorption rates on filter membranes has been found for aminoglycosides, glycopeptides, fluroquinolones and polymyxins [3]. The reports for ciprofloxacin and tigecycline, though, are still lacking in number. Nevertheless, there are good reasons to believe that both of these drugs may be adsorbed on filter membranes: ciprofloxacin, as it belongs to the same group as levofloxacin, found to undergo adsorption, and tigecycline owing to its ability to adsorb to organic compounds not only via the mechanism of electrostatic attraction, but also through metal bridging by forming ternary complexes [4, 5]. The previous studies in vitro conducted by the authors of this paper involving crystalloids demonstrated significant adsorption of tigecycline on polyacrylonitrile (PAN) membrane used in CRRT [6].

The material of which filter membrane and its surface are made constitutes one of the main factors determining the degree of drug adsorption. In the last decade, with a view to increasing the effectiveness of cytokine adsorption, filters with PAN were introduced into clinical use, with the membrane being additionally covered with positively charged polyethyleneimine (PEI) [7]. The change resulted in the reduced surface negative charge of the PAN membrane and thus can affect the adsorption of antibiotics with positively charged molecules, such as aminoglycosides [8, 9]. Another recent technological modification was increasing surface area in filters used for CRRT. In an attempt to enhance the effectiveness of the procedure, together with an increase in blood flow rate through the filter its surface area was increased, from values below 1.0 m^2^ up to 2.0 m^2^. The latter change may also have a substantial effect on the level of drug adsorption [10].

Another important factor playing a role in adsorption of antibiotics on filter membrane is the presence of blood proteins and cellular blood components [11, 12]. The presence of proteins and erythrocytes decreases the free fraction of a drug available for binding to filter membrane, but on the other hand proteins bound to antibiotics may also be adsorbed on the filter [11]. There may be two stages of serum protein adsorption on the filter surface: the first one involving adsorption of proteins with high molecular weight on the filter surface (for example albumins) and the second stage consisting in adsorption of proteins inside the membrane (proteins of low or medium molecular weight). Both of these mechanisms may have an effect on adsorption of antibiotics [13]. Owing to the fact that some of the studies on antibiotic adsorption were conducted with crystalloid solutions, they did not deal with the phenomena mentioned above.

The study presented here is a continuation of our research published in October 2019 and its aim was the assessment of in vitro adsorption of antibiotics representing different therapeutic groups (vancomycin, gentamicin, ciprofloxacin and tigecycline) on filters of large surface area: PEI PAN and polysulfone (PS) filters using porcine blood as a model close to in vivo conditions.

## Materials and methods

### In vitro study circuit

**Fig.1:**
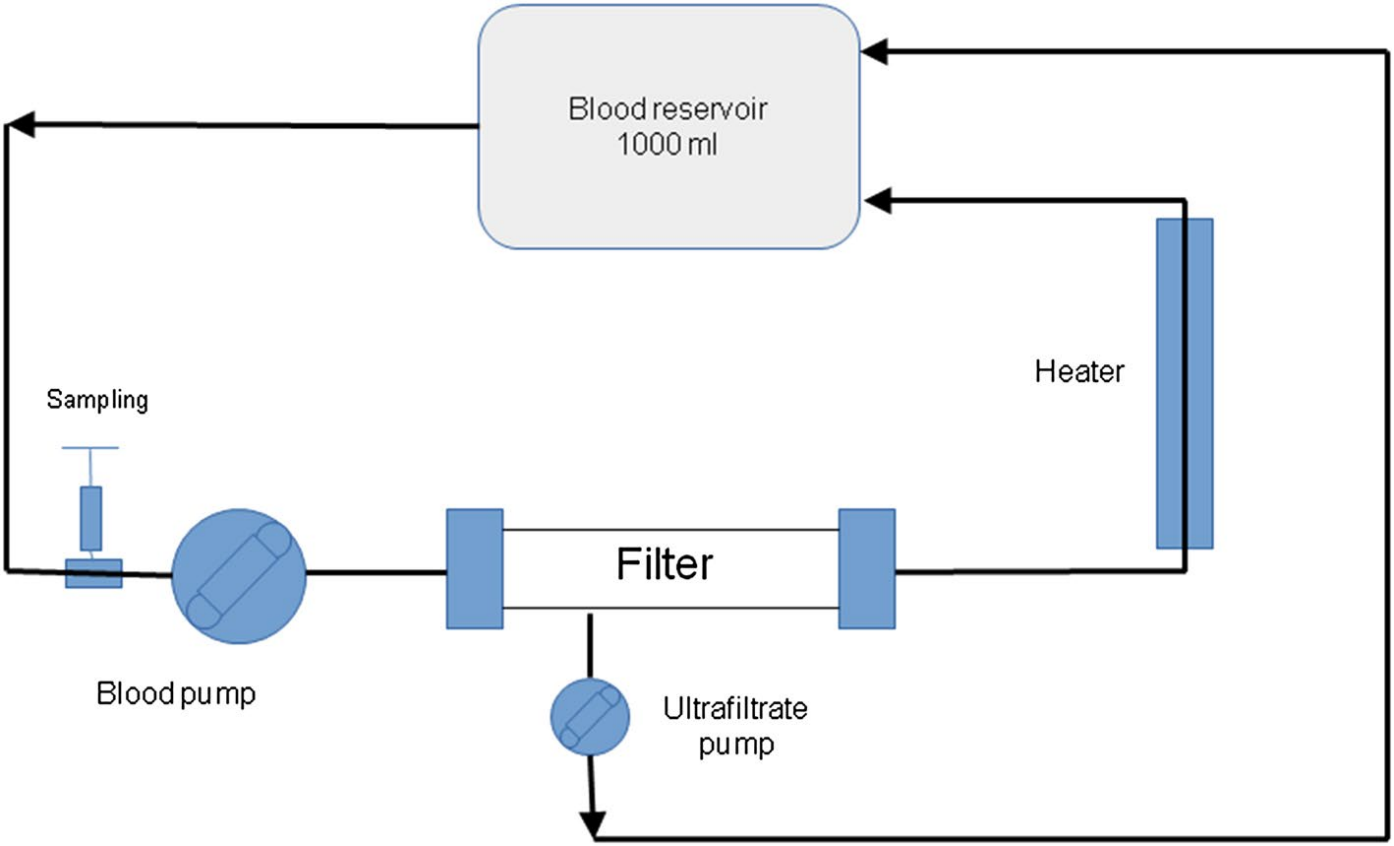
Diagram showing continuous veno-venous hemofiltration circuit used in the in vitro study of vancomycin, gentamicin, ciprofloxacin and tigecycline adsorption on polyacrylonitrile covered with polyethyleneimine and polysulfone membranes

The aim of the study was to assess adsorption on two types of membranes: PEI PAN membrane of AN69ST filter (Baxter) with surface area of 1.5 m^2^ and PS membrane of AV 1000 filter (Fresenius Medical Care, Germany) with surface area of 1.8 m^2^ using a circuit for continuous veno-venous hemofiltration (CVVH) filled with blood. The assessment of adsorption was conducted on a machine used in the clinical setting (Multifiltrate, Fresenius Medical Care, Germany) with a CVVH kit by the same provider, with either AN69ST or AV 1000 filter connected into the kit (Fig. 1). The total volume of the blood compartment of the kit and the filter was approximately 200 ml. Adsorption was assessed in the CVVH circuit with blood flow rate of 100 ml/min and ultrafiltration rate of 600 ml/h. Before the study was commenced the circuit was filled with 0.9% saline solution without antibiotic. Next a reservoir was attached, containing porcine blood with antibiotic dissolved in it. After the CVVH was started, the first 200 ml of the fluid (0.9% saline solution) was removed. During the test the ultrafiltration fluid was continuously returned to the reservoir containing blood with antibiotic. A fluid warmer (Hotline,Smith Medical, UK) was used to keep the temperature of the recirculating fluid in the return line between 35 and 37 °C (Fig. 1). Prior to the beginning of the test the blood was buffered with a bicarbonate solution to obtain pH > 7.15 before antibiotic was added. Anticoagulation was achieved by adding 30 ml of 7.4% sodium citrate. The study assessed adsorption of vancomycin in the dose of 1000 mg (Edicin Sandoz GmbH, Austria), gentamicin in the dose of 400 mg (Gentamycin; Krka, Slovenia), ciprofloxacin in the dose of 400 mg (Ciprofloxacin Kabi; Fresenius Kabi, Germany) and tigecycline in the dose of 100 mg (Tygacil; Pfizer Limited, Great Britain), which are standard doses used in clinical practice. The volume of blood-filled reservoir was 1010 ml for vancomycin, gentamicin and tigecycline, and 1200 ml for ciprofloxacin. The higher volume for ciprofloxacin was caused by the fact that the drug is available as 2 mg/ml solution, while the other antibiotics are available as powder which were dissolved in 10 ml of water. For each filter type and antibiotic three study cycles were conducted.

Blood samples (3 ml) to determine antibiotic concentrations were taken at minutes 0, 5, 15, 30, 45, 60, 90 and 120 from the pre-blood pump port of the CVVH circuit.

To assess possible spontaneous degradation of the drug in the solution there was an additional reservoir prepared for each antibiotic, containing blood with the drug, which was identical to the one described above, but which was not connected to the CVVH circuit. The temperature of the blood was maintained at 35–37 °C. Also from this reservoir samples were taken at minute 0, 5, 15, 30, 45, 60, 90 and 120, counting from the time the solution was prepared. The results of the measurements obtained from the reservoir which was not undergoing CVVH constituted the control group.

The samples were centrifuged at 3000 rpm immediately after they had been taken to separate plasma. The obtained plasma was then frozen at –80 °C and, still frozen, was delivered to a laboratory for antibiotic concentrations to be determined.

### Drug analysis

For analysis of tigecycline and ciprofloxacin, high performance liquid chromatography was coupled with mass spectrometry and fluorescence detection, respectively. These analyses were performed using methods previously described by Onichimowski et al. [6]. Tigecycline was extracted from plasma samples according to Jasiecka et al. except for the use of tigecycline-d9 as an internal standard (instead of minocycline) [14]. Ciprofloxacin was extracted according to Ziółkowski et al. [15]. Because of changes in the extraction procedure (matrix type, ciprofloxacin instead enrofloxacin) both methods were revalidated with regard to linearity, precision, accuracy, total recovery and specificity, and for tigecycline, the matrix effect was also examined according to FDA guidance [16].

To test linearity, four repetitions of calibration curves (10 points each) were performed. The calibration curve ranged from 10.0 to 400.0 μg/ml for ciprofloxacin and from 1.0 to 150.0 μg/ml for tigecycline. In all analyses, linearity was high, as shown by *r*^2^ values of 0.99 for all curves, and the deviation of each calibration point was less than ± 15% from the nominal concentration. Based on analysis of four concentrations (six replicates at each concentration), which was repeated over 3 days, the accuracy of the ciprofloxacin and tigecycline analyses was 4.74–9.11% and 3.21–7.85%, respectively, and the precision of these analyses was 3.96–9.97% and 4.11–7.85%, respectively. The total recovery of ciprofloxacin was about 93%, and that of tigecycline, approximately 50%. The plasma did not show any endogenous peaks in retention time with either drug. For tigecycline, neither ionization suppression nor ionization enhancement were relevant.

Total drug adsorption was calculated on the basis of the fall in the drug concentration in the solution. The calculation for vancomycin, gentamicin and tigecycline was done according to the following formula:

$$Drug \, adsorption \, = \, C_{0} \left( {{{\mu g} \mathord{\left/ {\vphantom {{\mu g} {ml}}} \right. \kern-\nulldelimiterspace} {ml}}} \right) \times 1010\;ml{-}C_{120} \times 1010\;ml.$$

For ciprofloxacin, the formula was as follows:

$$Drug \, adsorption \, = \, C_{0} \left( {{{\mu g} \mathord{\left/ {\vphantom {{\mu g} {ml}}} \right. \kern-\nulldelimiterspace} {ml}}} \right) \times 1200\;ml{-}C_{120} \times 1200\;ml,$$

where *C* stands for concentration.

Total drug adsorption was only calculated for the results which were statistically significant.

### Statistical analysis

In statistical analysis (mean ± SD; *n* = 3) drug concentration values at particular time points were compared. Statistical analysis was performed using two-tailed, unpaired Student’s *t* test (GraphPad Prism 3.1; Graphpad Software, San Diego, CA, USA) and *p* < 0.05 was considered statistically significant.

## Results

For vancomycin the mean hematocrit values in the blood used in the study for samples with PEI PAN membrane, PS membrane and from the control were 33.6, 33.0 and 36.0% respectively, for gentamicin 37.3, 34.6 and 35%, for ciprofloxacin 24.6, 24.3 and 25%, while for tigecycline 35.0, 33.0, and 35.0%, respectively. Lower hematocrit values obtained for ciprofloxacin resulted from the fact that the drug was added to blood in the form of a solution of 200 ml volume. No statistically significant differences in hematocrit values were found between the samples for particular antibiotics.

**Fig. 2:**
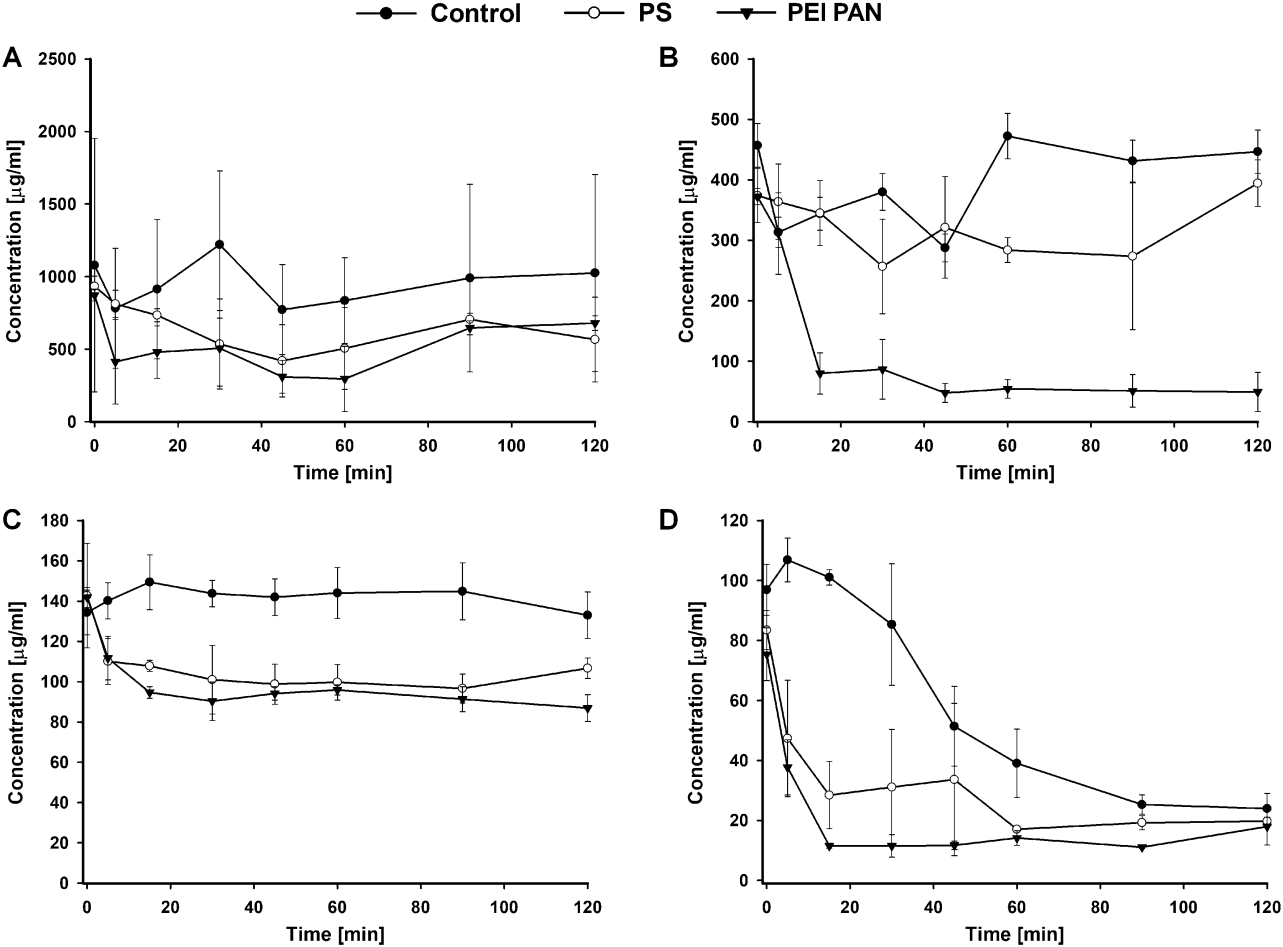
Vancomycin (**a**), gentamycin (**b**), ciprofloxacin (**c**) and tigecycline (**d**) concentrations (± SD, *n* = 3) in the porcine blood during continuous veno-venous hemofiltration (CVVH*) *in vitro; *control* concentrations in blood reservoir without CVVH, PS concentrations during CVVH with polysulfone membrane, PEI-PAN—concentrations during CVVH with polyacrylonitrile treated polyethyleneimine membrane

In the part of the study which was to determine spontaneous degradation of the drugs in the solution no significant fall in the drug concentrations over time was found for vancomycin and gentamicin (Fig. 2a, b). Their concentrations after 120 min did not differ statistically from their initial concentrations. In the case of ciprofloxacin the concentration remained unchanged for 120 min; its value, however, was over twofold lower than would be expected after solving 400 mg of the drug in 1200 ml (blood and drug mixture), that is 140 vs. 333 mg/l (Fig. 2c). In the case of tigecycline the concentration began to decrease slowly, beginning with minute 30, down to 24.7% of the initial concentration after 120 min (Fig. 2d).

In the assessment of vancomycin adsorption in CVVH circuit a statistically significant decrease in the level of vancomycin was observed when compared to the initial value T_0_ on both types of membrane: at minute 15 (*p* < 0.05) and from minute 45 to 120 (*p* < 0.05) of the study for PEI PAN membrane, and at minute 15, 45 and 90 (*p* < 0.05) of the study for PS membrane (Fig. 2a). For both membranes at minute 90 and 120 of the study readsorption occurred and the level of the drug in blood returned to about 80% of the initial values. In comparison to control blood the decrease was significant only for PEI PAN membrane with a statistically significant difference from minute 15 to 90 (*p* < 0.05). The values of vancomycin adsorption on PEI PAN and PS membranes were 195.26 and 370.34 mg, respectively.

In the assessment of gentamicin adsorption in CVVH circuit there was a statistically significant decrease in the drug concentration noted for PEI PAN membrane only (both in comparison to the initial *T*_0_ value and to the value in control blood–*p* < 0.001) (Fig. 2b). The drug concentration was observed to fall rapidly already in the first 15 min of the test, going down to 21.46% of the initial gentamicin level. At minute 120 the drug concentration was as low as 13% of its initial value. The adsorption level for PEI PAN membrane was 326.23 mg. No readsorption was observed.

In the assessment of ciprofloxacin adsorption in CVVH circuit a significant decrease in the drug concentration in comparison to initial value T_0_ as well as to the concentrations in the control blood was observed, both for PEI PAN and PS membranes (*p* < 0.001 and *p* < 0.05 respectively) (Fig. 2c). A significant fall occurred as early as minute 5 of the test for PEI PAN membrane and minute 15 for PS membrane. No statistically significant differences in concentration falls were observed between the membranes. The level of adsorption was 64.95 mg for PEI PAN membrane, and 43.24 mg for PS membrane. No readsorption was observed.

The assessment of tigecycline in CVVH circuit in comparison to the control group a statistically significant fall in the drug concentration was observed both for PEI PAN and PS membranes (*p* < 0.001 and *p* < 0.05 respectively) (Fig. 2d). A significant decrease in the drug concentration both in comparison to the initial values and to the control group occurred in minute 5 of the test. Tigecycline concentrations in CVVH circuit remained low throughout the test for both membrane types. From minute 45 statistically significant differences in tigecycline concentration between CVVH-treated blood and control blood disappeared. However, leveling of the concentrations was not the result of readsorption, but of the drop in tigecycline concentration in the control sample. The adsorption level for PE PAN membrane was 58 mg and for PS membrane 64.36 mg.

## Discussion

Optimum dosing of antibiotics is an essential element of effective therapy in patients with severe infections or septic shock. Critically ill patients develop significant pathophysiological changes affecting drug pharmacokinetics such as increased capillary permeability, fluid retention or hypoalbuminemia, which may lead to an increase in volume of distribution of some drugs and thus to a decrease in their blood concentrations [17]. Moreover, in patients requiring CRRT additional drug elimination occurs and one of its important mechanisms could be adsorption on filter membrane in the CRRT circuit. This phenomenon depends on many factors such as electric charge of the filter membrane, membrane material as well as its structure, an electric charge of a drug molecule and the degree of plasma protein binding. The filter membrane charge is most likely to play a large role in the level of drug adsorption, as was demonstrated by recent papers by Wan et al. and Economou et al. Both groups used similar experimental design, but the first group used PAN membranes and observed that 46–48% of ticarcillin was adsorbed within 20 min of administration, while the second group using PES membranes observed a maximum level of ticarcillin adsorption not exceeding 15% [18, 19]. In a study by Economou et al. the level of piperacillin adsorption depended mostly the concentration of the drug in the experimental circuit and was irrespective of the blood flow rates used [19]. In the studies by Shiraishi et al. an addition of albumins to the solution reduced the adsorption of teicoplanin on the filters of CRRT circuit despite significant interactions between albumin and filter membrane. Although the albumins, together with antibiotic bound, underwent adsorption on the filter, the fact that antibiotic binds to albumins, and so reduces a free drug fraction available for adsorption on the membrane, resulted in the reduction of total adsorption [2]. In turn, in their studies on anidulafungin, Kolbinger et al. demonstrated that, when solved it in a crystalloid solution, its proportion undergoing adsorption on the filter membrane was about 99%, while it was only 60% following the addition of albumins, with just 35% of the antibiotic undergoing absolute adsorption when administered into blood. It suggested that in the studies of drug adsorption on filter membranes in CRRT circuits, the presence of cellular blood components should be considered [12]. Nussbaumer–Pröll et al. proved that in the presence of erythrocytes there is a fall in the activity of meropenem, ciprofloxacin and tigecycline [11].

The above factors considered, in this study, being a continuation to previous investigations involving the use of crystalloids Onichimowski et al. [6] porcine blood was used as a model most closely resembling in vivo conditions. The results obtained show that in a blood-filled CVVH circuit a reduction in vancomycin concentration occurred, both for PEI PAN and PS membranes. However, when adsorption of vancomycin was assessed with antibiotic used in the same dose (1 g) and CVVH circuit but with a crystalloid solution, adsorption was observed only on AN69ST membrane [6]. The proportion of vancomycin binding to albumins is relatively high at 30–55%, and the latter in turn easily bind to PS membranes [9]. In the present study, the presence of albumins could therefore result in significant adsorption on PS membrane. In the case of PEI PAN membrane the amount of vancomycin adsorbed was slightly greater in the presence of albumins (195.26 mg) when compared to the previous studies (181.88 mg) [6]. This means that binding of this drug on PEI PAN membrane is only to a small extent dependent on the presence of proteins and is in fact of electrostatic nature, although the presence of proteins may increase adsorption. The results obtained in the study are in line with the previous study results by Quale et al. which also demonstrated a slight increase in vancomycin adsorption on PAN membrane in the presence of proteins [20]. PEI on PAN membrane in AN69ST filters changes the adsorption properties of the membrane increasing adhesion of some complement components (C3a and C5a, factor D), β2macroglobulins, and also by reducing negative membrane charge [9, 21]. The analysis of the results from both studies shows that addition of PEI to PAN membrane did not have a considerable effect on adsorption of vancomycin. In the case of PS membranes the presence of proteins is necessary for adsorption to take place, and then it may even exceed 30%. The study by Tian et al. comparing the adsorption of vancomycin on polyamide, PS and PAN membranes in the mixture of blood with crystalloid solution showed that the adsorption on the latter was the greatest at 10.08 mg, which accounted for 28% of the administered drug dose. The adsorption on PS membrane, though, was considerably lower at 4.8 mg [22]. The sum total of adsorption was lower than in our study due to a significantly lower initial drug concentration in the solution (50 mg/L vs. about 1 g/L).

In the case of gentamicin a statistically significant adsorption was observed only for AN69ST circuit. The adsorption was rapid and irreversible and its value was almost 90% of the dose used (326.23 mg). In contrast to vancomycin no adsorption on PS membrane was observed despite the presence of proteins in the solution. This could be explained by a low proportion of gentamicin binding to blood proteins (< 10%) [23]. The results of our studies correlate with those reported by Lam et al. who investigated adsorption of gentamicin in blood-crystalloid mixture and demonstrated adsorption of this drug on PAN membrane to be 90% of the initial dose; it was also shown to be rapid and irreversible [24]. A similar, very high adsorption capacity of PAN filter was demonstrated by Tian et al. for amikacin and by Konfol et al. for tobramycin [25, 26]. Considering the fact that clinical efficacy of aminoglycosides depends on their reaching appropriately high maximum concentration in relation to minimal inhibitory concentration (C_max_/MIC), it does seem reasonable to stop CRRT for the time of drug infusion, so that the drug can achieve the required therapeutic concentration in blood. The fact of increased adsorption occurring on filter membranes may in turn be used to minimize the risk of adverse effects of aminoglycosides when high doses of these are used to treat infections caused by multidrug resistant strains. Other authors also suggest that adsorption of aminoglycosides on the filter membranes during CRRT should be considered in the dosage regimens [3, 7, 23].

The findings for ciprofloxacin were particularly interesting. In our previous study Onichimowski et al. [6], where we assessed adsorption of ciprofloxacin in CVVH circuit in vitro in the crystalloid solution, no significant irreversible adsorption of the drug was observed, regardless of the type of filter used. In the present study, due to the use of porcine blood, there were significant changes observed already in the control group. The drug concentration remained unchanged for 120 min, but its value was over twofold lower than expected (140 vs. 333 mg/L). Ciprofloxacin is characterized by a very good penetration to tissues [27, 28]. Moreover, the studies by Nussbaumer-Pröll et al. showed that in the presence of erythrocytes the antibacterial activity of ciprofloxacin falls, which might be explained by its penetration to red blood cells or it is binding to their surface [11]. The study also demonstrated that the addition of human erythrocytes to standard growth media (Mueller Hinton Broth II) caused a decrease in extracellular concentration of drug, while no such phenomenon was observed for meropenem or tigecycline. The distribution of ciprofloxacin and ofloxacin into erythrocytes was previously demonstrated by Colino et al. [29]. In the present study a decrease in ciprofloxacin concentration was seen in blood-filled CVVH circuit both for PEI PAN and PS membranes; adsorption in comparison to initial values was 45% and 30%, respectively. Significant levofloxacin adsorption on PAN filters was also observed by other authors [30, 31]. Sufficient literature data concerning ciprofloxacin adsorption are still lacking; however, there are reports on substantial extrarenal clearance of this drug during CRRT, which necessitated the use of high doses to achieve adequate concentrations in blood [3, 32]. Adsorption on filters may partially explain this phenomenon.

Tigecycline is a new drug which belongs to glycylcyclines, recently introduced into clinical practice. It is a lipophilic drug of a very high volume of distribution (7–9 L/kg) binding to proteins at 71–89%, showing atypical nonlinear plasma protein binding dependent on divalent metal ions [33, 34]. In the present study, in the blood-filled CVVH circuit a decrease in tigecycline concentration was observed both for PEI PAN as well as PS membrane. Adsorption was rapid and irreversible and was found to be nearly at 80% of the drug dose used. In the study which assessed adsorption of tigecycline used in the same dose (100 mg) and CVVH circuit in crystalloid solution, adsorption occurred only on PEI PAN membrane Onichimowski [6]. The proportion of tigecycline binding to albumins is very high, and the latter in turn easily adsorb on PS membranes [9]. The presence of albumins in this study could cause significant adsorption also on PS membrane. In our study we noted a gradual decrease in tigecycline concentration in control blood not undergoing CVVH, beginning with minute 30 of the test. The concentration of the drug fell to 24.7% of the initial concentration at minute 120 of the test. This effect may be explained by a very high volume of drug distribution and penetration of tigecycline to erythrocytes. Nussbaumer-Pröll et al. showed a reduction in antibacterial activity of the drug in the presence of erythrocytes [11]. Of date, there are no reports from studies on adsorption of tigecycline on filter surface during CRRT in blood-filled circuits we could compare the results of our studies with. However, considering the properties of the drug, i.e., its ability to adsorb on the surfaces of organic substances, its electrostatic properties as well as metal-binding properties resulting in the formation of ternary complexes, adsorption of this drug on the surface of CRRT filter should not be surprising [4].

### Limitations

In the study presented here standard doses of drugs used in clinical practice were applied. This made it possible to assess filter adsorption capacity for the full range of drug amount found in patient’s blood in vivo. However, owing to the fact that the volume of blood in which the drug was solved was 1 L, the concentration of the drug substantially exceeded those observed in blood in vivo. Drug concentration does not have to, but still may exert an influence on the amount of the drug adsorbed [31].

In our study anticoagulation in CVVH circuit was achieved by decreasing the concentration of ionized calcium; sodium citrate was added according to the rules applied in regional citrate anticoagulation (RCA) during CRRT. This strategy made the concentration of calcium ions and probably also other divalent ions in the mixture very low. In the case of tigecycline this fact may have an effect on both the volume of direct adsorption on filter membranes as well as on the degree of binding to blood proteins and indirect adsorption [34, 35]. At present RCA is the most commonly used method of anticoagulation; however, considering the above, to assess adsorption of tigecycline during CRRT with an alternative method of anticoagulation, i.e. anticoagulation with heparin, further studies are required.

For PS filters the comparison of results for adsorption without and in the presence of blood proteins showed that presence of the latter considerably increases adsorption. Critically ill patients often present with hypoproteinemia and hypoalbuminemia and, to assess the adsorption capacity of PS membranes, more studies should be conducted involving various levels of blood proteins.

## Conclusion

Optimum dosage of antibiotics is an essential part of treatment in patients who develop severe infections or septic shock. Introducing CRRT into the therapy for these patients considerably affects elimination of antibiotics from blood. Apart from convection and diffusion an important mechanism of antibiotic elimination seems to be adsorption on filters. Our studies demonstrated that in full blood adsorption of antibiotics, both hydrophilic such as vancomycin and gentamicin and lipophilic such as ciprofloxacin and tigecycline may be big enough to be of clinical significance. The comparison of adsorption during CVVH for antibiotics solved in full blood and for antibiotics solved in the crystalloid solution shows a significant role of blood proteins, especially for PS membranes, due to their strong affinity to bind proteins. This means there is a need for further research which would determine the level of adsorption in relation to blood protein concentration, as critically ill patients tend to present with considerable abnormalities regarding this parameter. In the case of tigecycline the fact that its adsorption as well as the degree of its binding to proteins depend on the concentration of divalent metal ions, the studies on its adsorption during CRRT should be conducted separately for citrate and heparin anticoagulation. Due to the fact that, as demonstrated in our study, adsorption on filter membranes used during CRRT is a phenomenon that does take place, a detailed quantitative determination of its range in clinical setting may confer really benefits in optimization of antibiotic dosing in critically ill patients.
